# Srtio_3_-Based Composites for Photocatalytic Panels in Solar Hydrogen Production

**DOI:** 10.3390/molecules30183699

**Published:** 2025-09-11

**Authors:** Aibol Baratov, Alexey Dikov, Lyubov Dikova, Tamara Aldabergenova, Timur Zholdybayev, Egor Maksimkin, Kira V. Tsay

**Affiliations:** 1 Department of Materials Science, Nanotechnology and Engineering Physics, Satbayev University, Almaty 050032, Kazakhstan; 2 Institute of Nuclear Physics, Almaty 050032, Kazakhstan; dikov@inp.kz (A.D.);

**Keywords:** photocatalysis, SrTiO_3_:Al, cocatalysts, nano-SiO_2_

## Abstract

This study investigates photocatalytic cells based on cocatalyst-loaded SrTiO_3_:Al and nano-SiO_2_ as a porous binder, immobilized on frosted glass. Comprehensive analysis confirmed the successful incorporation of aluminum into SrTiO_3_, increasing oxygen vacancy concentration and enhancing charge transfer. The deposition of RhCr_2_O_3_ and CoOOH cocatalysts significantly improved photocatalytic activity, boosting hydrogen and oxygen evolution rates to 3.8401 and 1.6319 mmol g^−1^ h^−1^, respectively. The introduction of nano-SiO_2_ increased hardness (0.23–0.25 GPa) and Young’s modulus (5.27–5.40 GPa), reinforcing structural integrity. The development of efficient photocatalytic panels requires a multifaceted strategy that considers chemical, mechanical, and optical properties together with stability, durability, and energy efficiency. Future research should focus on optimizing these key parameters to enhance system performance for industrial applications.

## 1. Introduction

The development of photocatalysts represents one of the most promising directions in modern science [1,2]. Their applications span a wide range of areas, including water and air purification [3], the degradation of organic pollutants [4], and photocatalytic hydrogen generation from water under solar irradiation [5,6]. Despite the active advancement of photocatalysts, their large-scale implementation is hindered by several factors, including low photocatalytic activity, high charge carrier recombination rates, and insufficient mechanical stability [7,8,9]. A key research focus is the development of new photocatalytic materials with enhanced efficiency and stability [10,11,12]. Among these materials, SrTiO_3_ is of particular interest due to its high chemical and thermal stability, excellent charge carrier mobility, and reduced recombination probability [13,14,15,16]. Despite its favourable properties, pristine SrTiO_3_ typically exhibits limited photocatalytic activity due to poor visible light absorption and high recombination rates, which can be significantly improved by cocatalyst loading and doping strategies [17,18]. To enhance the efficiency of SrTiO_3_, various modification strategies are actively being explored, including Al doping and the decoration with Rh-, Cr-, and Co-based nanoparticles [19,20,21]. It has been demonstrated that incorporating aluminum nanoparticles into the SrTiO_3_ structure reduces the recombination rate of photogenerated charge carriers [22]. According to several studies [23,24,25], the modification of SrTiO_3_ with aluminum nanoparticles enhances charge separation efficiency, increases light absorption, and improves the photocatalytic performance of the material. In addition, silicon dioxide (nano-SiO_2_) has emerged as an effective porous binder that enhances mechanical strength and improves the immobilisation of photocatalyst layers on solid supports.

For the large-scale implementation of photocatalytic materials, a promising approach is their integration into structural panels [26,27], that operate under solar irradiation [28,29]. However, the successful industrial deployment of photocatalytic panels requires overcoming several key limitations related to the mechanical stability of materials, their environmental durability, the efficiency of photocatalyst deposition, and their integration into existing energy systems [30,31]. One of the critical challenges is enhancing the mechanical strength of photocatalytic panels, which requires a clearer understanding of mechanical stress—namely, the internal force per unit area arising from external loads, thermal expansion, or structural deformation. Such stress can lead to fatigue, microcracking, or delamination over time, especially under cyclic loading. Previous studies have reported that mechanical stress can significantly impair the structural integrity and long-term performance of photocatalytic materials [32,33]. In addition, external factors such as intense ultraviolet radiation, temperature fluctuations, and moisture exposure can accelerate these effects, further highlighting the need for in-depth research on mechanical stability. Another crucial aspect of improving the efficiency of such systems is optimizing the methods for depositing the photocatalyst onto the substrate. Enhanced immobilization should minimize the overlap of active sites and ensure their maximum accessibility. Moreover, specialized protective coatings with hydrophobic or chemically resistant properties can extend the lifespan of photocatalytic panels [34,35]. Thus, the development of photocatalytic panels requires further research to enhance their stability, optimize photocatalyst deposition techniques, improve mechanical strength, and adapt them to real-world operating conditions.

This study examines photocatalytic cells based on modified SrTiO_3_ (cocatalyst-loaded STO:Al) and nano-SiO_2_, which serves as a porous binder, deposited on the surface of frosted glass. Their chemical, mechanical, and optical properties, as well as durability and photoefficiency, are investigated. The influence of composition and structural features on the mechanical properties of the panels is analyzed, allowing for an assessment of their potential for further integration into technological and energy solutions.

## 2. Experimental Part

### 2.1. Materials

Titanium(IV) oxide, anatase (TiO_2_, particle sizes < 25 nm, 99.7%), strontium nitrate Sr(NO_3_)_2_ ≥ 98%), silicon dioxide (SiO_2_ < 99.99%), aluminium oxide (Al_2_O_3_, particle sizes < 50 nm, 99.8%), rhodium(III) chloride hydride (RhCl_3_·6H_2_O, Rh 38–40%), cobalt (II) nitrate hexahydrate (Co(NO_3_)_3_·6H_2_O, ≥98%), and methylene blue (C_16_H_18_ClN_3_S·H_2_O, dye content, ≥82%) were purchased from Sigma–Aldrich (St. Louis, MO, USA). Oxalic acid ((COOH)_2_·2H_2_O, >98%), strontium chloride hexahydrate (SrCl_2_·6H_2_O, 99.7%), potassium chromate (K_2_CrO_4_, 99.5%), were purchased from Laborpharma (Almaty, Kazakhstan). All the chemicals were used without any additional treatment.

### 2.2. Synthesis of SrTiO_3_ and SrTiO_3_:Al

SrTiO_3_ powder was synthesized using the methodology detailed in our previous works [36,37,38]. Briefly, TiO_2_ was added to an aqueous solution of Sr(NO_3_)_2_ in a molar ratio of 1:1. Under vigorous stirring, 0.4 M (COOH)_2_·2H_2_O was introduced into the solution to maintain the pH level at 6–7. The resulting precipitate was dried for 16 h and then calcined at 900 °C for 1 h. Aluminum doping of the obtained SrTiO_3_ powder was carried out using a flux treatment method. For this purpose, Al_2_O_3_ and SrCl_2_ were added to the SrTiO_3_ powder as fluxing agents in a molar ratio of 1:0.02:10. All components were mixed in an agate mortar and placed in a crucible for heating at 1150 °C for 10 h. The samples were then subjected to ultrasonic treatment, washed five times with distilled water, and dried for 20 h. The resulting sample is called STO:Al.

### 2.3. Photodeposition of Cocatalysts

The dual cocatalysts Rh/Cr_2_O_3_ and CoOOH were deposited on the STO:Al photocatalyst using the photodeposition method, as detailed in our previous works [24,39]. Briefly, 0.1 g of STO:Al was dispersed in distilled water, sonicated, and irradiated under a 10 W UV lamp. RhCl_3_⋅6H_2_O, K_2_CrO_4_, and Co(NO_3_)_3_ solutions were sequentially introduced, with irradiation after each step. The samples were then thoroughly washed and dried at 60 °C. The final cocatalyst concentrations were 0.1% Rh, 0.05% Cr, and 0.05% Co. For conciseness, the cocatalyst-photodeposited samples are referred to as Cocat/STO:Al.

### 2.4. Application of Photocatalyst Powders on the Substrate

The photocatalyst powders were immobilized onto frosted glass substrates with an area of 25 cm^2^. Before immobilization, the glass substrates were treated with a concentrated with 0.1 M H_2_SO_4_ solution and cleaned with an alcohol solution to improve wettability and ensure strong binding of the photocatalytic particles. The photocatalyst layers were formed using the drop-casting method. A mixture of 25 mg of photocatalyst powder, 25 mg of nano-SiO_2_, and 500 μL of distilled water was prepared to fabricate laboratory-scale photocatalytic cells with a working area of 25 cm^2^. The suspension was sonicated for 10 min to break up agglomerates, then applied dropwise (50 μL per drop) onto frosted glass substrates, ensuring uniform distribution, and dried at 70 °C. This process was repeated 10 times, followed by annealing in air at 350 °C for 1 h.

### 2.5. Characterization

Diffraction measurements were performed using the D8 ADVANCE universal diffractometer (Bruker, Germany) in the Bragg–Brentano θ-θ geometry, equipped with a copper anode tube (wavelength 1.5406 Å). The operating parameters were set to 40 kV and 40 mA, with a 2θ range of 5–80°, a step size of 0.02°, and a scanning speed of 0.5 s per step. The morphology and elemental composition of the samples were analyzed using a scanning electron microscope (Zeiss Crossbeam 540, Oberkochen, Germany) at an accelerating voltage of 5–20 kV, equipped with an energy-dispersive X-ray spectrometer (EDX) (INCA X-Sight, Oxford Instruments). Surface characterization of the synthesized photocatalytic panels was conducted using a Hitachi TM4000 scanning electron microscope, equipped with an elemental analysis attachment and secondary electron (SE) and backscattered electron (BSE) detectors. X-ray photoelectron spectroscopy (XPS) was carried out using a VG Microtech Multilab 3000 spectrometer (VG Microtech Ltd., London, UK) with Mg and Al X-ray sources to analyze the valence states and elemental composition of the samples. The C1s peak at 284.8 eV was used as a reference for binding energy (BE) calibration. Ultraviolet-visible diffuse reflectance spectra (UV-Vis DRS) were recorded on a Perkin Elmer Lambda 35 spectrophotometer in the 200–800 nm range. Mechanical properties were measured using nanoindentation with the “NanoScan-4D Compact” instrument based on the irreversible indentation method (ISO 14577). A Berkovich three-sided diamond pyramid was used as the indenter, with a load of 8 mN. The load application time was 5 s, with a holding time of 0.5 s at the specified load. The analysis of the load–displacement curves was performed using the Oliver–Pharr method [37,38], which enables the determination of coating hardness (H, GPa) and Young’s modulus (E, GPa).

### 2.6. Photocatalytic Measurements

The photocatalytic activity of immobilized photocatalysts was evaluated in a photochemical reactor (Shanghai Leewen Scientific Instrument Co., Ltd., Shanghai, China) under irradiation from a 10 W mercury lamp(The illumination intensity is adjusted to 12 W/m^2^). The photocatalytic cell (with an active area of 25 cm^2^ and a catalyst mass of 25 mg) was placed in a sealed quartz reactor containing 50 mL of distilled water. Before the experiment, the reaction system was purged with argon to create an inert atmosphere. The generated gaseous products were analyzed using a “CHROMOS 1000” gas chromatograph, equipped with three packed columns (3 mm in diameter) filled with NaX and PORAPAK Q sorbents, directly connected to the reactor.

## 3. Results and Discussion

### 3.1. Characterization of Samples

#### 3.1.1. Microstructural and Morphological Properties

**Figure 1:**
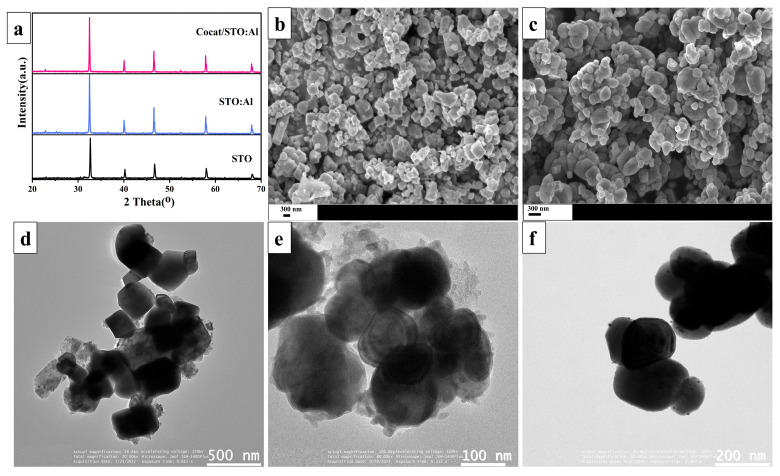
(**a**) XRD patterns of obtained photocatalysts; SEM images of samples based on (**b**) STO and (**c**) STO:Al; TEM images of samples based on (**d**) STO, (**e**) STO:Al and (**f**) Cocat/STO:Al.

Figure 1a presents the X-ray diffraction (XRD) patterns of the synthesized SrTiO_3_-based samples. Diffraction peaks corresponding to the (110), (111), (200), (211), and (220) planes are observed at 32.40°, 40.50°, 46.50°, 58.50°, and 68.50°, respectively, indicating the cubic phase of SrTiO_3_ (JCPDS #35–0734). The high intensity and sharpness of the peaks suggest a high degree of crystallinity in the material. No phase reflections corresponding to the added elements (Al, Rh, Cr, Co) are detected, likely due to their low concentration and weak crystallinity. It is also noted that flux-assisted doping improves the crystallinity of SrTiO_3_. Electron microscopy techniques were employed to investigate the morphology of the synthesized samples. According to SEM and TEM data (Figure 1b,d), the STO particles exhibit a cubic shape with sizes ranging from 200 to 400 nm. After flux-assisted Al doping (Figure 1c,e), the STO:Al facets become smoother due to the selective adsorption of Cl^−^ on {111}, which lowers surface energy and promotes the reduction of {100} facets [40,41,42]. This treatment also leads to the formation of more uniform particles with an average size of ~150 nm. Figure 1f illustrates the presence of RhCr_2_O_3_ and CoOOH-based cocatalysts (5–15 nm) on the surface of STO:Al. Studies indicate that pristine STO and STO:Al exhibit lower activity compared to the modified samples, as the absence of cocatalysts limits the formation of active sites [24,43,44]. Cocatalysts not only provide active sites for photocatalytic reactions but also enhance charge transfer through the formation of a Schottky junction, thereby reducing electron–hole recombination and improving photocatalytic efficiency [45,46].

Figure S1 in the Supplementary Material presents SEM images of glass substrates before (a) and after (b) sandblasting treatment. The sandblasting process ensures sufficient adhesion for the effective fixation of the photocatalyst powder on the glass substrate surface. Figure S2 displays SEM images of the photocatalytic layer formed by the drop-casting method on a textured glass substrate. As shown in Figure S2d, the SrTiO_3_:Al-based powder layer is uniformly distributed due to the use of nano-SiO_2_ as a binder. This not only enhances adhesion to the substrate but also promotes the formation of a porous structure, optimizing the penetration of light, water, and the generated gases. The average thickness of the resulting coating is approximately 20 µm, which is optimal for efficient photocatalytic reactions (Figure S2d) [27].

#### 3.1.2. Surface Chemistry and Optical Properties

**Figure 2:**
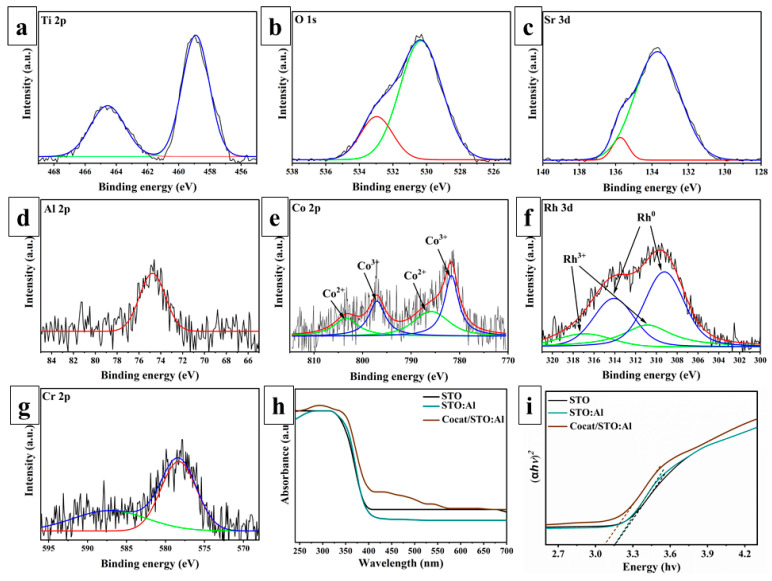
High-resolution XPS spectrum of Cocat/STO:Al sample for (**a**) Ti 2p, (**b**) O 1s, (**c**) Sr 3d, (**d**) Al 2p, (**e**) Co 2p, (**f**) Rh 3d and (**g**) Cr 2p; (**h**) absorption spectra and (**i**) values of the bandgap of synthesized samples.

XPS analysis was performed on STO:Al samples with deposited cocatalysts RhCr_2_O_3_ and CoOOH to study their chemical composition and surface states (Figure 2a–g). In the Ti 2p spectrum (Figure 2a), peaks at 458.9 and 464.5 eV are observed, corresponding to Ti^4+^ [19]. The HR-XPS O 1s spectrum (Figure 2b) shows two main signals: 530.3 eV, attributed to lattice oxygen (O^2−^), and 532.9 eV, corresponding to adsorbed oxygen (O_ads) [39]. It was established that Al doping increases the concentration of O_ads, which positively impacts the catalytic properties of the material compared to pristine STO [33]. The Sr 3d spectrum (Figure 2c) shows characteristic doublet peaks typical for Sr^2+^ in the perovskite structure. The Al 2p peak at 74.7 eV (Figure 2d) confirms the incorporation of Al into the STO lattice. It is known that Al^3+^ effectively suppresses undesirable Ti^3+^ defects and increases the concentration of oxygen vacancies, reducing recombination of photogenerated charges and stabilizing the defective structure [47,48].

Furthermore, high-resolution XPS spectra confirmed the presence of cocatalysts on the surface of STO:Al. The Rh 3d spectrum (Figure 2f) exhibits peaks at 309.2/314.1 eV and 310.9/316.9 eV, corresponding to metallic Rh^0^ and oxidized Rh^3+^ species, respectively, with the amount of Rh^3+^ being relatively small [49]. The Rh 3d spectrum exhibited Rh^3+^ species along with traces of metallic Rh^0^ (Figure 2d). Metallic Rh acts as an electron sink by forming Schottky junctions with STO:Al, facilitating rapid electron capture, while Rh^3+^ contributes to interfacial redox cycling, accelerating the hydrogen evolution reaction (HER). Additionally, the presence of Cr^3+^ species from Cr_2_O_3_ (Figure 2e) was confirmed, which stabilizes Rh nanoparticles by forming a protective shell and enhances their durability under illumination. The Cr 2p spectrum (Figure 2g) displays peaks at 578.3 eV (Cr 2p_3_/_2_) and 587.1 eV (Cr 2p_1_/_2_), corresponding to Cr^3+^ [50]. The Co 2p spectrum (Figure 2e) contains components of both Co^2+^ and Co^3+^, with characteristic peaks at 803.4/785.9 eV and 797.0/781.7 eV, respectively. These species may originate not only from Co(III)OOH but also from oxide phases such as CoO. These observations indicate that the cocatalysts likely comprise a mixture of different oxidation states and chemical compositions, which serve as oxidative sites for hole accumulation and promote the oxygen evolution reaction (OER) through efficient utilization of photogenerated h^+^ [20].

The optical properties of STO, STO:Al, and Cocat/STO:Al samples were investigated by UV–vis spectroscopy. While STO and STO:Al primarily absorb light in the UV region (up to 400 nm), the Cocat/STO:Al composite shows extended absorption up to ~430 nm (Figure 2h). The Tauc plot analysis of the (αhν)^2^–hν dependence revealed a bandgap of 3.15 eV for STO and STO:Al, and 3.07 eV for Cocat/STO:Al (Figure 2i). Aluminum doping effectively suppresses Ti^3+^ defects but has no significant effect on the band gap width [21], whereas cocatalyst deposition enhances light absorption and charge separation efficiency, thereby reducing recombination losses.

### 3.2. Nanomechanical Testing: Nanoindentation

**Figure 3:**
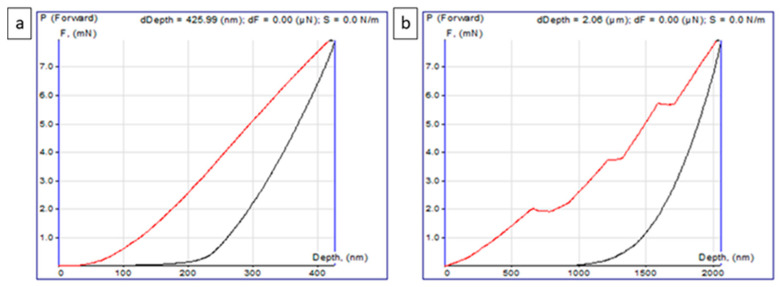
Loading–unloading curves obtained during nanoindentation: (**a**) classical shape of the curve without discontinuities on the loading branch; (**b**) curve with characteristic step-like features on the loading branch (red line), while the unloading curve (black line) remains smooth.

To evaluate the hardness and elastic modulus of the photocatalyst layers, nanoindentation tests were conducted. The load–displacement analysis allowed the determination of the mechanical properties of the coatings using the force law method according to Oliver and Pharr [51]. Young’s modulus, which characterizes the resistance to changes in interatomic distances, was calculated from the slope of the “loading–unloading” curve [52]. The tests were performed at 25 °C, and the values of hardness and Young’s modulus were determined as the average of five measurements, excluding the extreme values. The “loading–unloading” curves for measuring the mechanical properties typically exhibited a classical shape (Figure 3a), although, in some cases, steps were observed on the loading curve (red line). The unloading curves (black line), however, remained stable (Figure 3b). The presence of steps indicates localized areas of coating looseness, where the indenter penetrated to a depth of approximately 2 µm. Data from such areas were excluded from the analysis as artifacts. Figure 3 shows that the maximum indentation depth was approximately 425.99 nm, which is equivalent to about 2% of the average film thickness. All curves exhibited similar behavior in terms of plastic deformation, with the indentation imprint remaining stable, without signs of elastic relaxation.

Table 1 presents the results of the mechanical analysis of thin films. The STO:Al@nano-SiO_2_ coating demonstrates a hardness in the range of 0.18–0.20 GPa, which is attributed to its dense structure and the absence of cracks. A similar trend is observed for the Young’s modulus (3.77–4.89 GPa). The Cocat/STO:Al@nano-SiO_2_ sample shows a slight increase in hardness values (0.23–0.25 GPa) and Young’s modulus (5.27–5.40 GPa), indicating that the introduction of co-catalysts does not lead to significant morphological changes. For comparison, it has been reported in the literature [53] that the addition of ZnO nanoparticles to epoxy coatings increases their hardness and Young’s modulus by reducing the free volume in the matrix. Similarly, the introduction of nano-SiO_2_ in Cocat/STO:Al also contributes to the improvement of mechanical properties. In particular, the study [53] found that the maximum hardness (0.205 GPa) and Young’s modulus (4.95 GPa) were achieved with the addition of 5% SiO_2_ and 2% ZnO, while exceeding this concentration led to a decline in properties due to nanoparticle agglomeration. In our study, a similar trend is observed for Cocat/STO:Al, which demonstrates hardness values (0.23–0.25 GPa) and Young’s modulus (5.27–5.40 GPa). Thus, the obtained results confirm the patterns previously described in the literature and emphasize the effectiveness of modifying Cocat/STO:Al with nano-SiO_2_ to improve mechanical strength without significant morphological changes [54,55].

### 3.3. Photocatalytic Performance

**Figure 4:**
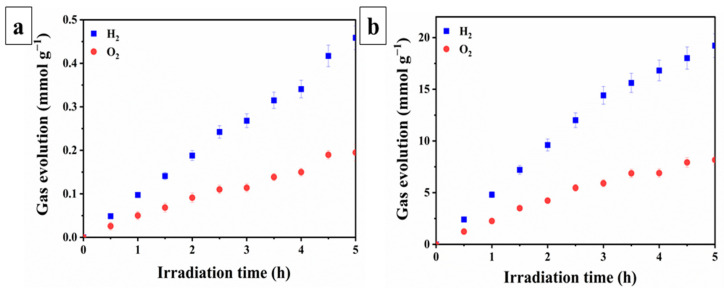
Water splitting performance of photocatalytic sheets based on (**a**) STO:Al and (**b**) Cocat/STO:Al.

The photocatalytic activity of the samples immobilized on a glass substrate was investigated in the photocatalytic water splitting reaction for hydrogen (H_2_) and oxygen (O_2_) evolution. As shown in Figure 4, which presents the results of photocatalytic water decomposition using the STO:Al photocatalyst without (Figure 4a) and with (Figure 4b) photodeposited cocatalysts, the efficiency of the samples significantly depends on the presence of cocatalysts. The average H_2_ and O_2_ evolution rates for the STO:Al-based photocell were 0.0916 ± 0.0039 and 0.0389 ± 0.0013 mmol g^−1^ h^−1^, respectively, whereas the Cocat/STO:Al exhibited much higher values—3.8401 and 1.6319 mmol g^−1^ h^−1^. The relatively low efficiency of STO:Al compared to Cocat/STO:Al can be attributed to the accelerated charge transfer and separation of photogenerated charges, which reduces recombination, as well as the formation of active sites upon the deposition of cocatalysts via photodeposition. This significant improvement directly correlates with the XPS results, which confirmed the presence of Rh^3+^, Cr^3+^, and Co^3+^ species on the STO:Al surface. These oxidation states act as catalytically active centers, facilitating charge transfer processes: Rh and Cr species promote electron trapping and reduction reactions, while Co^3+^ sites acceler-ate hole utilization in oxidation reactions. Such cooperative effects suppress charge recombination and create efficient redox pathways, thereby enhancing overall photo-catalytic performance. However, in comparison to powder counterparts, the investigated samples show lower efficiency. Specifically, powder Cocat/STO:Al under similar conditions exhibited H_2_ and O_2_ evolution rates of 11.04 and 4.69 mmol g^−1^ h^−1^, respectively [24]. The decrease in activity after the photocatalyst was applied to the substrate is related to partial overlap of active particles. Nevertheless, the use of nano-SiO_2_ as a binder helps mitigate this effect by creating micropores that facilitate the contact of water and light with the active surface of the photocatalyst [27,28,56].

## 4. Conclusions and Future Prospectives

This study investigated photocatalytic cells based on a cocatalyst-loaded STO:Al photocatalyst and nano-SiO_2_ as a porous binder, immobilized on the surface of matte glass. Comprehensive analysis confirmed the successful incorporation of aluminum into SrTiO_3_, as confirmed by XPS analysis. This increased the concentration of oxygen vacancies and improved charge transfer. The deposition of co-catalysts (RhCr_2_O_3_ and CoOOH) significantly enhanced the photocatalytic activity by effectively separating charge carriers and creating active sites. Mechanical property analysis revealed that the addition of nano-SiO_2_ increased the hardness (0.23–0.25 GPa) and the Young’s modulus (5.27–5.40 GPa), confirming the suitability of its use for strengthening the structural integrity of photocatalytic films. Photocatalytic tests of the samples immobilized on glass substrates showed a significant increase in activity after the deposition of co-catalysts: the hydrogen and oxygen evolution rates increased to 3.8401 and 1.6319 mmol g^−1^ h^−1^, respectively. The development of efficient photocatalytic panels requires a comprehensive approach that includes the study of their chemical, mechanical, and optical properties, as well as the evaluation of stability, strength, and energy efficiency. Large-scale testing of such systems has already been conducted. A striking example is the work of Professor Kazunari Domen’s group, which demonstrated the safe operation of photocatalytic panel reactors with an effective area of 100 m^2^ [23]. These systems successfully operated for several months, demonstrating the possibility of large-scale water splitting, hydrogen and oxygen collection, and separation, which confirms the promising nature of the technology. Further research should focus on studying the influence of external factors and optimizing key aspects to improve the efficiency and stability of such systems.

For successful scaling of the technology to industrial applications, the following key aspects must be considered:

Mechanical Stability: Further investigation of the strength characteristics of photocatalytic panel cells during long-term operation, including fatigue resistance, thermal expansion, and resistance to mechanical stresses.

Environmental Impact: Studying the effects of prolonged UV exposure, temperature fluctuations, and humidity on the structural stability of photocatalytic films.

Optimization of Photocatalyst Application: Improving immobilization techniques to minimize overlap of active sites and enhance the catalyst’s accessibility for the reaction.

Development of Protective Coatings: The use of hydrophobic or chemically resistant materials to increase the lifespan of photocatalytic panels under real-world operating conditions.

Energy Efficiency and Integration: Exploring the potential for combining photocatalytic panels with other renewable energy sources (solar panels, thermoelectric modules) to increase the overall efficiency of the system.

Thus, the conducted research demonstrates significant progress in the development of photocatalytic materials. However, further work should focus on improving their stability, mechanical strength, and adaptability to real-world operating conditions, which is a crucial step toward their implementation in large-scale applications.

## Figures and Tables

**Table 1:** Hardness values and modulus of elasticity calculated for photocatalytic coatings.

№	Sample	E, GPa	H, GPa
1.1	STO:Al	3.77 ± 2.70	0.18 ± 0.02
1.2	STO:Al	4.89 ± 2.22	0.20 ± 0.04
2.1	Cocat/STO:Al	5.40 ± 1.14	0.25 ± 0.0.4
2.2	Cocat/STO:Al	5.27 ± 1.07	0.23 ± 0.06

## Data Availability

Data are contained within the article.

## Supplementary Materials

The following supporting information can be downloaded at: https://www.mdpi.com/article/10.3390/molecules30183699/s1, Figure S1: SEM images of the glass substrate surface (a) before and (b) after sandblasting; Figure S2: SEM images of a photocatalytic layer formed by drip casting onto a textured glass substrate: (a,c) images at various magnifications, (b) energy dispersive X-ray spectroscopy (EDX) analysis showing the elemental composition, (d) layer thickness of a powder co-catalyst based on modified STO:Al.
