# Supplementary material for: Srtio3-Based Composites for Photocatalytic Panels in Solar Hydrogen Production
Source: Molecules. 2025 Sep 11;30(18):3699. doi: 10.3390/molecules30183699 (PMC12472357; doi:10.3390/molecules30183699)
Supplement: Supplementary file 1 [file molecules-30-03699-s001.zip › molecules-3845393-supplementary.pdf]

SSrtio3-Based Composites for Photocatalytic Panels in Solar Hydrogen Production

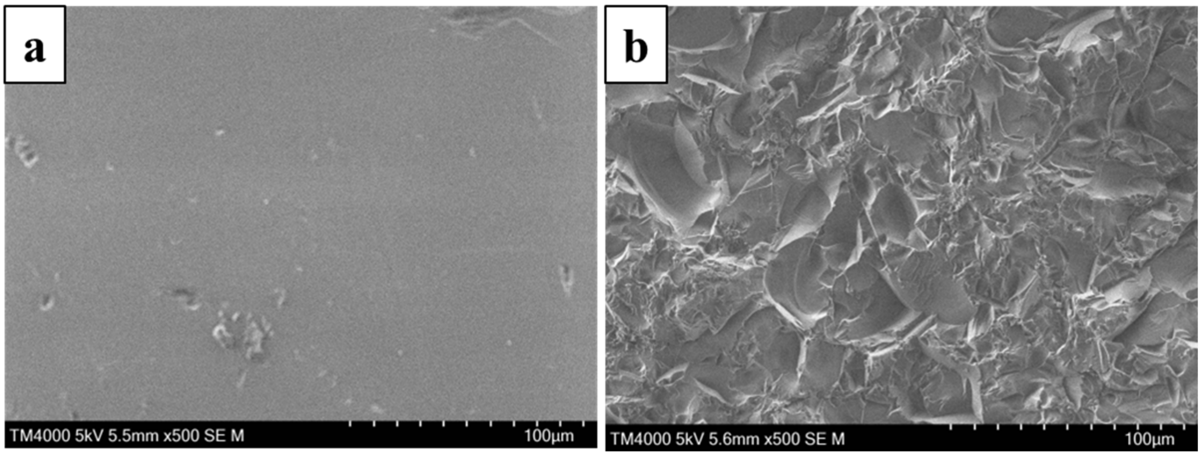

Figure S1 SEM images of the glass substrate surface (a) before and (b) after sandblasting

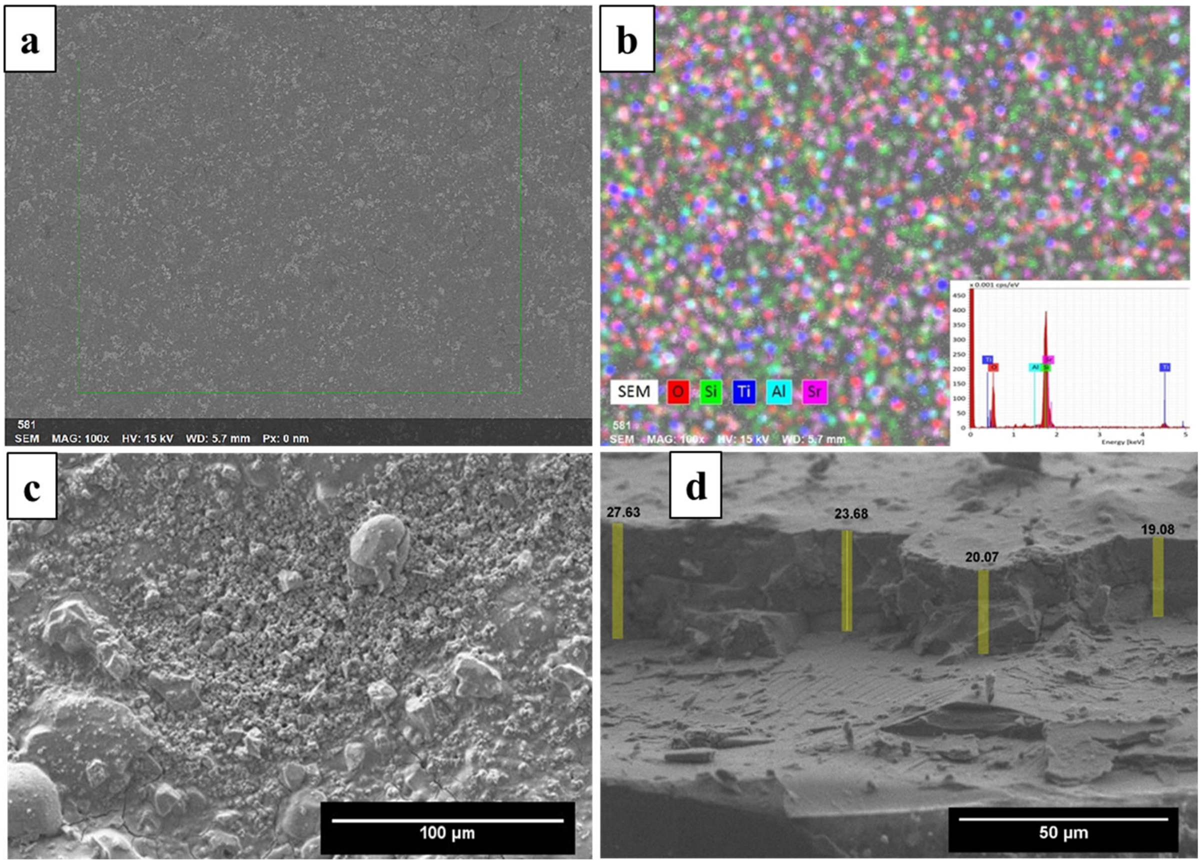

Figure S2 SEM images of a photocatalytic layer formed by drip casting onto a textured glass substrate: (a-c) images at various magnifications, (b) energy dispersive X-ray spectroscopy (EDX) analysis showing the elemental composition, (d) layer thickness of a powder co-catalyst based on modified STO:Al
